# Financial development during COVID-19 pandemic: the role of coronavirus testing and functional labs

**DOI:** 10.1186/s40854-021-00226-4

**Published:** 2021-01-29

**Authors:** Muhammad Khalid Anser, Muhammad Azhar Khan, Khalid Zaman, Abdelmohsen A. Nassani, Sameh E. Askar, Muhammad Moinuddin Qazi Abro, Ahmad Kabbani

**Affiliations:** 1 School of Public Administration, Xi’an University of Architecture and Technology, Xi’an, 710000 China; 2 Department of Economics, University of Haripur, Haripur Khyber Pakhtunkhwa, Pakistan; 3 Department of Management, College of Business Administration, King Saud University, P.O. Box 71115, Riyadh, 11587 Saudi Arabia; 4 Department of Statistics and Operations Research, College of Science, King Saud University, P.O. Box 11451, Riyadh, 11587 Saudi Arabia; 5 Department of Management, Aleppo University, Aleppo, Syria

**Keywords:** Financial development, COVID-19 pandemic, Infected cases, Testing capacity, Robust least square estimator, Innovation accounting matrix

## Abstract

The outbreak of the SARS-CoV-2 virus in early 2020, known as COVID-19, spread to more than 200 countries and negatively affected the global economic output. Financial activities were primarily depressed, and investors were reluctant to start new financial investments while ongoing projects further declined due to the global lockdown to curb the disease. This study analyzes the money supply reaction to the COVID-19 pandemic using a cross-sectional panel of 115 countries. The study used robust least square regression and innovation accounting techniques to get sound parameter estimates. The results show that COVID-19 infected cases are the main contributing factor that obstructs financial activities and decrease money supply. In contrast, an increasing number of recovered cases and COVID-19 testing capabilities gave investors confidence to increase stock trade across countries. The overall forecast trend shows that COVID-19 infected cases and recovered cases followed the U-shaped trend, while COVID-19 critical cases and reported deaths showed a decreasing trend. Finally, the money supply and testing capacity show a positive trend over a period. The study concludes that financial development can be expanded by increasing the testing capacity and functional labs to identify suspected coronavirus cases globally.

## Introduction

**Fig. 1:**
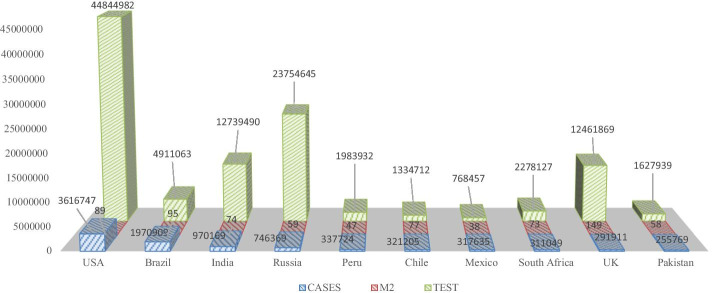
COVID-19 infected cases, financial development, and testing scores. *Source*: Worldometer (2020, 16th July)

The control of excess money supply and credit creations through bank rates is considered one of the oldest instruments for monetary policy adoption by the central bank to re-settle economic issues (Auclert 2019). The adverse shocks to monetary policy spur various differential outcomes in lowering interest rates and increasing asset pricing (Schmidt 2020). Empowering governance and capability structure for sustainable innovative programs, support corporate vision towards eco-friendly production (Awan 2020). The COVID-19 pandemic has hampered economic activities and financial transactions, leading to high volatility in the stock prices (Fallahgoul 2020; Procacci et al. 2020). Increased COVID-19 infected cases and deaths globally has slowed down stock market indices and increased negative financial values (Albulescu 2020; Sapkota and Madai 2020). Brown and Rocha (2020) concluded that entrepreneurial activities have been affected during the current pandemic, which affects the debt market, causing a global financial crisis to emerge. The entrepreneurial equity investment in a given scenario is disturbed, and strategic policy intervention is required to overcome the rising debt, to minimize uncertainty in the entrepreneurial business. Ali et al. (2020) showed that the COVID-19 outbreak started in China, moved to Europe and then to the US, and created disorder to global financial activities, leading to effects in the latter phase, when COVID-19 reached the US. The effects of the pandemic even affected relatively safer commodities in the latter phases. Shehzad et al. (2020) discussed the opportunity to invest in the Asian market, instead of Europe and the USA, for better portfolio optimization during the COVID-19 pandemic. They critically analyzes the effects of a global financial crisis in 2008 (GFC) and the COVID-19 crisis on the world economies. Figure 1 shows the factual assessment of infected cases, money supply, and testing facility in the ten most affected countries.

Figure 1 illustrates that operational and testing lab capacity are higher in the USA, followed by Russia, India, UK, and Brazil, and cases increased at a tremendous rate in the USA, Brazil, India, and Russia. The money supply is higher in the UK and lowest in Mexico at the given point in time.

This study used four main factors for analyzing the COVID-19 pandemic (infected cases, reported deaths, recovered cases, and critical cases) and the money supply, while earlier studies were limited to a few variables (see, Goodell et al. 2020). Second, coronavirus testing capacity was used as a mediator between COVID-19 cases and money supply, which was not done in the earlier literature. Third, the money supply is used as a “response variable” and a proxy for financial development, affected by COVID-19 associated factors. The relationship between money supply and COVID-19 factors is verified by innovation accounting matrix and forecast evaluation indices to reach certain policy conclusions. This study proposes to satisfy the following research objectives:

(i) To examine the impact of coronavirus infected cases, reported deaths, critical cases, and recovered cases on money supply in a cross-sectional panel of 115 countries.
(ii) To evaluate the role of coronavirus testing capacity in improving financial development across countries, and
(iii) To analyze the forecast evaluation of the candidate variables over a time horizon.

These objectives have been verified through a cross-sectional regression estimator, innovation matrix, and forecast indices. The study has the following sections. The introduction is presented in Sect. 1. Literature review is in Sect. 2. Data & methodology are presented in Sect. 3. Section 4 discussed the results, while the final section concludes the study.

## Literature review

The number of scholarly works available on the COVID-19 pandemic shows its negative effect on every sector of the global world (Codagnone et al. 2020; Dzigbede and Pathak 2020). These adverse effects are not limited to healthcare casualties, but has also severely affected the financial sector, destabilizing investors' confidence in stock trading (Topcu and Gulal 2020; Ibn-Mohammed et al 2020; Phan and Narayan 2020). The sustainable development goals are lost their momentum during the pandemic (Heggen et al. 2020). Hence, there is a greater need to devise innovative policies to combat the increased social and environmental sufferings (Amankwah-Amoah 2020; Awan 2020; Heinonen and Strandvik 2020). The increased need for coronavirus testing capacities may shorten the learning curves by sharing knowledge and technological spillovers (Kumar et al. 2020). The technical absorptive capacity is not only helpful to mitigate negative environmental concerns, but can be further used in many healthcare interventions to reduce the risk of infectious diseases (Sarkar et al. 2020, Cheng et al. 2020, Kanwal and Awan 2020, Tromberg et al. 2020). The financial risk reporting based on generating algorithms can use to avoid bankruptcy, which required prudential economic policies to prevent it from systemic risk (Kou et al. 2014, 2019, 2021; Chao et al. 2019, 2020).

Specific literature is presented to observe financial volatility due to the COVID-19 pandemic across various economic settings. For instance, Topcu and Gulal (2020) found that stock markets negatively reacted because of shocks to real oil prices and currency exchange rates, due to increased COVID-19 infected cases. The stock market volatility is highest in emerging markets followed by the Middle East and South America; while Central & Eastern Europe is least affected. It is critical that governments minimize the financial crisis through various stimulus packages in order to reduce stock market volatility across countries. Al-Alwadhi et al. (2020) considered the Chinese stock market to analyze the vulnerability of COVID-19 infected cases and reported deaths on stock market returns across all companies, and found a negative correlation between them. The need for quick response time to contain coronavirus cases and reported deaths may help stabilize stock market trends. Li et al. (2020) found that the outbreak of the COVID-19 virus, considered the primary driver of stock market volatility, resulted in increased insurance premiums. The optimal guarantee may have been enhanced through capital regulations during the current pandemic. The sound financial activities are vital for resolving the conflicts between board members and insurer to settle their accounts efficiently. Mazur et al. (2020) used the USA to analyze the stock market performance in the wave of coronavirus outbreak and found high volatility in stock trading of real estate business stocks, petroleum products, and services stocks while observed positive stock trading in information technology-based products, healthcare products, and food stocks. The impacts found in different trading stocks needs more critical review to understand the investors’ centric approach.

Nicola et al. (2020) discussed the socio-economic consequences faced globally because of the COVID-19 pandemic such as, reduced workforce due to the lockdown; exacerbated unemployment because of closures of industries, educational institutes, and other corporate businesses; increased need for healthcare medical supplies; and increased demand for food items due to panic buying. Global strategic decision-making needs to develop resilient strategies to combat COVID-19. Waris et al. (2020) described the global efforts to reduce coronavirus infection. The study appreciated the efforts of Pakistan’s government that contained coronavirus cases by adopting rigorous measures to minimize the spread of coronavirus, that is, strict compliance to the Standard Operating Procedures (SOPs), designed special hospitals and healthcare facilities, increased laboratory testing capacity, and introduced multiple forms of lockdown initiatives. The efforts were highly valued that helps to formulate resilient healthcare policies for global prosperity. Fang and Meng (2020) emphasized the importance and need for clinical laboratories to detect infectious diseases (including COVID-19), at early stages to contain its spread. Vandenberg et al. (2020) suggested that continuously improving clinical diagnostic testing services, would not only support early detection of an infected person but can be used as a policy tool to optimize healthcare resources, to prevent further healthcare casualties globally. Oldekop et al. (2020) discussed four main economic factors affected by the COVID-19 pandemic. First, the global value chain has been disrupted due to restrictions to transportation systems, which led to severe consequences for essential healthcare medical supplies, food shortages, and manufacturing exports; second, digitalization, since the use of online working tools have increased because of working at home; third, public finance is now confined within the country and cannot be transformed into production workflows; and finally, climate change efforts to reduce global carbon emissions remains stagnant. These factors remain critical and need to be resolved with joint international collaboration.

Barbier and Burgess (2020) pointed out that sustainable development goals were barely achieved in many parts of the global world due to exacerbated COVID-19 infected cases. The low adaptability of innovative technologies and the absence of international support make developing countries severely vulnerable, affecting the nation's economic growth. The unexpected increase in the need for global healthcare infrastructure increased the strain on the already severe financial crises in developing countries. There is therefore, a need to pursue healthcare compatibility and innovative strategies to escape from the pandemic and the financial crisis. Yamey et al. (2020) stressed the need for the coronavirus vaccine, as it was the only way to control the pandemic. Study shows three imperatives for a possible coronavirus vaccine; first, the process for making the vaccine need to be quick; second, deployment at a massive scale; and third, ensuring global access to all. Priority must be given to healthcare workers and infected patients and then distribution to the non-infected cases on an equally.

Based on the stated literature, the study selected the main predictors of the study defined in the subsequent section.

## Data sources and methodological framework

The study used a broad money supply relative to GDP (denoted by M2) as a proxy variable for financial development served as a “regressand variable.” In comparison, the counts of coronavirus registered cases (denoted by CASES), death cases (denoted by DEATHS), recovered cases (denoted by RECOVERED), critical cases (denoted by CRITICAL), and coronavirus testing (denoted by TEST) served as “regressors.” The available data for the M2 variable is taken from World Bank (2020), whereas the data for COVID-19 variables are taken from Worldometer (2020, 16th July). The cross-sectional data is used as one point in time with a panel of 115 countries. Table 1 shows the list of sample countries for easy reference.

**Table 1:** List of sample countries

Total countries: 115	“USA, Brazil, India, Russia, Peru, Chile, Mexico, South Africa, UK, Pakistan, Turkey, Bangladesh, Colombia, Qatar, Egypt, Iraq, China, Indonesia, Sweden, Ecuador, Belarus, Kazakhstan, Philippines, UAE, Ukraine, Bolivia, Dominican Republic, Singapore, Poland, Afghanistan, Nigeria, Romania, Armenia, Guatemala, Honduras, Azerbaijan, Ghana, Japan, Algeria, Moldova, Serbia, Nepal, Morocco, Cameroon, Uzbekistan, Kyrgyzstan, Kenya, Australia, El Salvador, Costa Rica, Malaysia, North Macedonia, Senegal, Bulgaria, Bosnia and Herzegovina, Finland, Haiti, Tajikistan, Gabon, Madagascar, Luxembourg, Djibouti, Hungary, Greece, Albania, Thailand, Paraguay, Somalia, Equatorial Guinea, Maldives, Cuba, Slovakia, Iceland, Slovenia, Guinea-Bissau, Cabo Verde, Sierra Leone, Libya, Hong Kong, Yemen, Rwanda, Benin, Mozambique, Tunisia, Latvia, Niger, Zimbabwe, Liberia, Uganda, Cyprus, Uruguay, Georgia, Namibia, Andorra, Suriname, Jamaica, Malta, Angola, Syria, Botswana, Viet Nam, Mauritius, Comoros, Guyana, Burundi, Mongolia, Gibraltar, Bermuda, Brunei, Aruba, Barbados, Bhutan, Gambia, Macao, Belize”

The study used the following equation to assess the effects of COVID-19 pandemic on money supply during increased testing capacity and reported critical cases:

1 $$\begin{gathered} \ln (M2)_{i,t} = \alpha_{0} + \alpha_{1} \ln (CASES)_{i,t} + \alpha_{2} \ln (DEATHS)_{i,t} + \alpha_{3} \ln (RECOVERED)_{i,t} + \alpha_{4} \ln (CRITICAL)_{i,t} + \alpha_{5} \ln (TEST)_{i,t} + \varepsilon_{i,t} \quad \therefore \frac{\ln (M2)}{{\ln (CASES)}} < 0,\frac{\ln (M2)}{{\ln (DEATHS)}} < 0,\frac{\ln (M2)}{{\ln (RECOVERED)}} > 0,\frac{\ln (M2)}{{\ln (CRITICAL)}} < 0,\frac{\ln (M2)}{{\ln (TEST)}} > 0 \hfill \\ \hfill \\ \end{gathered}$$

Equation (1) shows that the money supply is expected to decline when there is an increase in the COVID-19 registered cases, deaths, and critical cases. An increase in testing capacity and patients recovered from coronavirus diseases is expected to expand more money supply across countries. The following hypotheses need to be tested for this to be conclusive:

### H1

COVID-19 registered cases, critical cases, and reported deaths will negatively impact money supply across countries*.*

### H2

Money supply is expected to have a positive response to increased COVID-19 recovered cases, and.

### H3

An increase in testing capacity for COVID-19 will positively impact the money supply.

These hypotheses are essential to analyze and devise strong global financial policies. The study used sequential steps for statistical analysis.

### Step–I: robust least square (RLS) estimator

The RLS estimator is the extended version of the simple least square regression, which addresses possible outliers of the dependent variable (by M-estimation technique), independent variables (by S- estimation technique) and simultaneously handle outliers from the regressand and regressors (by MM-estimation technique) from the model. The study used the M-estimation technique, as the money supply widely fluctuated during the COVID-19 pandemic. The M-estimation is abbreviated to "maximum likelihood estimator-like," magnifying the possible outliers in the regressand. This estimator is also known as the Huber-M estimator, as Huber (1973) proposed the way to minimize regressand outliers to transform robust least-square estimates. The S-estimation is abbreviated to “scale statistic” that addresses regressand outliers from the model. Rousseeuw and Yohai (1984) suggested that this estimation technique uses robust least-square estimates. Finally, the MM-estimator is proposed by Yohai (1987), considered the mixture of “M” estimator and “S” estimator, which handles the regressand and regressors outliers simultaneously, to provide more robust estimates (different from the simple least-square estimates).

### Step-II: innovation accounting matrix (IAM)

The innovation accounting matrix (IAM) procedure is based on the Vector autoregression (VAR) system's two innovation techniques, that is, impulse response function (IRF) and variance decomposition analysis (VDA). The IRF estimates show that systematic shocks about the *i*-th variable has translated into other system endogenous variables via the dynamic lag structure. The function traces the *i*-th shock of innovations on present and future endogenous variables during the VAR system. The VDA innovation technique is somehow different from the IRF estimates, as IRF estimates mark out the system shock one to other endogenous variables. At the same time, VDA provides the importance of the components shocks that separate it from the endogenous variable in random innovation in the VAR system.

### Step-III: forecast evaluation

The study further moves towards the forecast evaluation by four methods:

1. "Root Mean Square Error (RMSE),"
2. "Mean Absolute Error (MAE),"
3. "Mean Absolute Percentage Error (MAPE)," and
4. "Theil inequality coefficient."

These methods predict future deviation in individual variables where one can assess the candidate variables' direction over a time horizon.

## Results and discussion

Table 2 shows the descriptive statistics of the variables. The mean value of money supply shows that the selected cross-sectional countries have more than 64% of its money supply relative to its GDP. The maximum total number of coronavirus registered cases, reported deaths, and recovered patients are about to reach 3,616,747, 140,140 and 1,645,962, respectively with a mean value of 102,928, 3537 and 61,299 respectively. The critical cases are about to reach 16,459 with a mean value of 453. The full testing service is at 90,410,000 with an average value of 2,080,156. The description of the variables is further used in the regression estimator.

**Table 2:** Descriptive statistics

VDA estimates	VDA estimates	VDA estimates	VDA estimates	VDA estimates	VDA estimates	VDA estimates
Mean	64.419	102,928	3537	61,299	453	2,080,156
Maximum	386.139	3,616,747	140,140	1,645,962	16,459	90,410,000
SD	49.769	412,127	15,830.980	220,446.700	1983.428	9,978,908
Skewness	3.347	6.878	7.088	5.733	6.324	7.422
Kurtosis	19.524	54.590	57.087	37.720	45.955	61.899

*Source*: Worldometer (2020, 16^th^ July) and World Bank (2020). Note: M2 shows broad money supply, CASES show COVID-19 registered cases, DEATHS show COVID-19 death cases, RECOVERED shows COVID-19 recovered cases, CRITICAL shows COVID-19 critical cases, and TEST show COVID-19 testing capacity

Table 3 shows the robust least square regression estimates and found that COVID-19 infected cases substantially decrease money supply, due to investors' low confidence in stock market trading, which ceased economic activities across countries. The elasticity estimates confirmed that there was a less elastic relationship between the two variables; if there is a 1% increase in the infected cases, money supply decreases by 0.390 percentage points. Increase in COVID-19 testing capacity and COVID-19 recovered cases, both increases the investors’ confidence in continuing economic activities, which positively affects the money supply across countries. The elasticity estimates show that if the increase in recovered cases is 1%, the money supply increases by 0.359%. On the other hand, if the increase in testing capacity is 1%, the money supply increases by 0.232%. These results confirm the need for controlling COVID-19 through increased testing capacity and unified global policies. The results are in line with the earlier studies; Baker et al. (2020) found the adverse effects of the COVID-19 pandemic on a stock market performance that possibly be subsidized by expansionary economic policies. Ashraf (2020) confirmed the negative association between COVID-19 infected cases (and reported deaths) and stock market returns that may lead to more severe over time. Haroon and Rizvi (2020) confirmed that the immense disruption in the equity market arose because of coronavirus related panic-laden news across the globe.

**Table 3:** Robust least square estimates

Dependent variable: ln(M2)				
Variable	Coefficient	SE	z-Statistic	Prob
ln(CASES)	− 0.390340	0.145110	− 2.689966	0.0071
ln(CRITICAL)	0.007461	0.045988	0.162243	0.8711
ln(DEATHS)	− 0.082494	0.077710	− 1.061568	0.2884
ln(RECOVERED)	0.358547	0.127427	2.813745	0.0049
ln(TEST)	0.232845	0.055028	4.231390	0.0000
C	1.958477	0.579399	3.380188	0.0007
Robust statistics				
R^2^	0.166807	Adjusted R^2^	0.103687	
Scale	0.484890	Deviance	0.235119	
Rn-squared statistic	47.97638	Prob(Rn-squared stat.)	0.000000	

‘ln’ shows natural logarithm, M2 shows broad money supply, CASES show COVID-19 registered cases, DEATHS show COVID-19 death cases, RECOVERED shows COVID-19 recovered cases, CRITICAL shows COVID-19 critical cases, and TEST show COVID-19 testing capacity

The results of the study further connected with the findings of the subsequent studies that concluded that financial trading is affected due to increase COVID-19 infected cases (see, Erdem 2020; Baek et al. 2020; Chia et al. 2020). These studies highlighted the need to subsidize the financial and healthcare sector to marginalize coronavirus's adverse effects in economic and business processes.

Table 4 shows the IRF estimates and found that an increase in the number of COVID-19 critical cases, registered deaths, and recovered cases substantially decline money supply over a time horizon. The increase in the testing capacity under the suggested WHO guidelines, gives investors confidence to resume economic activities. In contrast, the increasing number of critical cases and reported deaths will deter investors from stock trades economic activities resume.

**Table 4:** IRF estimates

Response of ln(M2)						
Months	ln(M2)	ln(CASES)	ln(CRITICAL)	ln(DEATHS)	ln(RECOVERED)	ln(TEST)
September 2020	− 0.081765	0.089245	− 0.046740	− 0.199513	− 0.141159	0.100580
October 2020	0.082794	0.059305	− 0.119642	− 0.169229	− 0.056978	0.135813
November 2020	0.067628	0.081026	− 0.069807	− 0.131688	− 0.048519	0.123965
December 2020	0.098690	0.073576	− 0.060632	− 0.135930	− 0.097395	0.101724
January 2021	0.074980	0.072148	− 0.064648	− 0.155917	− 0.105412	0.103957
February 2021	0.089666	0.072536	− 0.084623	− 0.149722	− 0.067994	0.120638
March 2021	0.084261	0.078691	− 0.075475	− 0.141624	− 0.062926	0.117800
April 2021	0.082537	0.076289	− 0.064024	− 0.144353	− 0.088953	0.107160
May 2021	0.083375	0.073653	− 0.070595	− 0.151179	− 0.093733	0.108972

‘ln’ shows natural logarithm, M2 shows broad money supply, CASES show COVID-19 registered cases, DEATHS show COVID-19 death cases, RECOVERED shows COVID-19 recovered cases, CRITICAL shows COVID-19 critical cases, and TEST show COVID-19 testing capacity

Table 5 shows the VDA estimates and found that reported deaths will negatively shock the capital market causing a decline in investors’ behavior towards stock trading; hence, the money supply is expected to decline from September 2020 to May 2021. The results for testing capacity, recovered cases, registered cases, and critical cases, will be 11.508%, 7.031%, 5.057%, and 5.038%, respectively. The results show that reported deaths will be the primary predictor of financial development across countries.

**Table 5:** VDA estimates

Variance decomposition of LNM2:							
Period	SE	ln(M2)	ln(CASES)	ln(CRITICAL)	ln(DEATHS)	ln(RECOVERED)	ln(TEST)
September 2020	0.730966	85.02804	1.490629	0.408863	7.449837	3.729274	1.893360
October 2020	0.780594	75.68494	1.884322	2.707693	11.23269	3.802952	4.687405
November 2020	0.812652	70.52407	2.732716	3.236178	12.98988	3.865297	6.651859
December 2020	0.847079	66.26541	3.269542	3.490804	14.53051	4.879474	7.564254
January 2021	0.882484	61.77686	3.680853	3.752984	16.50954	5.922606	8.357161
February 2021	0.916970	58.17375	4.034943	4.327654	17.95710	6.035329	9.471224
March 2021	0.947479	55.27853	4.469051	4.687984	19.05354	6.093993	10.41690
April 2021	0.977078	52.69369	4.812020	4.837623	20.09932	6.559189	10.99816
May 2021	1.007749	50.21954	5.057744	5.038375	21.14500	7.031139	11.50821

‘ln’ shows natural logarithm, M2 shows broad money supply, CASES show COVID-19 registered cases, DEATHS show COVID-19 death cases, RECOVERED shows COVID-19 recovered cases, CRITICAL shows COVID-19 critical cases, and TEST show COVID-19 testing capacity

**Fig. 2:**
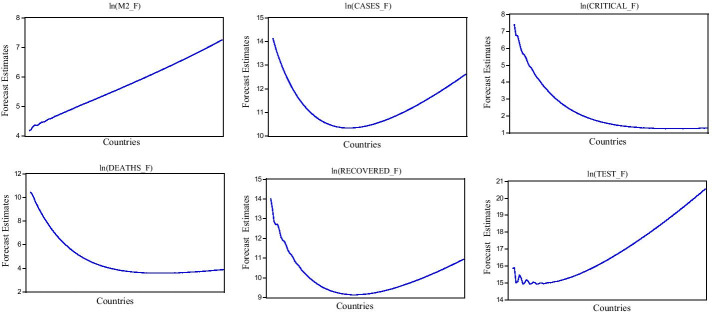
Overall forecast trend. Note: 'ln' shows natural logarithm, 'F' shows forecasted value, M2 shows broad money supply, CASES_F shows COVID-19 registered cases, DEATHS show COVID-19 death cases, RECOVERED shows COVID-19 recovered cases, CRITICAL shows COVID-19 critical cases, and TEST show COVID-19 testing capacity

Table 6 shows the forecast evaluation of the studied variables. The four suggested forecast estimators are used to assess the trend of the variables over a time horizon. The higher value of RMSE and MAE is found in the M2 variable followed by CASES, TEST, and DEATHS. In contrast, CASES have a higher forecast value in MAPE, followed by M2, DEATHS, and CRITICAL. Finally, the lowest value of RECOVERED is found in the Theil index, while the higher value of CASES is found in the same index. The given forecast evaluation shows the candidate variables' smooth curves that can be seen in Fig. 2.

**Table 6:** Forecast evaluation

Variables	Included observations	RMSE	MAE	MAPE	Theil
ln(M2)	115	7.384	7.317	64.627	0.480
ln(CASES)	115	6.551	6.416	349.374	0.559
ln(CRITICAL)	78	2.508	2.020	42.387	0.262
ln(DEATHS)	109	3.268	2.732	48.744	0.290
ln(RECOVERED)	113	2.953	2.102	20.804	0.159
ln(TEST)	108	6.117	5.054	28.470	0.210

Note: ‘ln’ shows natural logarithm, M2 shows broad money supply, CASES shows COVID-19 registered cases, DEATHS shows COVID-19 death cases, RECOVERED shows COVID-19 recovered cases, CRITICAL shows COVID-19 critical cases, and TEST show COVID-19 testing capacity

Figure 2 shows the overall forecast trend based on four suggested forecast indices. The candidate variables' smooth curves give some directions of variables over a time horizon. The money supply is expected to increase over a time horizon because of resuming economic activities after easing restrictions. The U-shaped curve is expected to decline initially in COVID-19 cases and peaks later on. A similar U-shaped trend is found in the COVID-19 recovered cases over a time horizon. The COVID-19 critical cases and reported deaths will substantially decline due to increased testing capacity across countries. The expansionary economic policies will help financial sector if testing capacity is not increased rapidly and the cases are not reduced swiftly.

## Conclusions and policy implications

This study assessed the financial volatility in global stock trading during the COVID-19 pandemic, and at the same time, the role of coronavirus testing and operational labs in building investors’ confidence through ex-ante and ex-post analysis, using cross-sectional data from 115 countries. The earlier studies were limited to either ex-post analysis or simulation to show ex-ante analysis. The study results found that coronavirus infected cases are considered the main factor that has impeded financial activities, while recovered cases and increased testing capacity together with functional labs, support financial development across countries. The innovation accounting matrix suggested that an increase in the coronavirus critical cases and reported deaths are likely to negatively impact financial development which can be improved by increased testing capacity and functional labs. The U-shaped forecast trends are visible in the coronavirus infected cases and recovered cases, while the substantial decline is likely to exhibit in the critical cases and reported deaths. Money supply and testing capacity both are likely to increase over a period of time.

The COVID-19 pandemic unprecedented increase in healthcare expenditures negatively impacted global economic activities. The nationwide lockdown, social distancing, transport and travel restrictions imposed many adverse effects on countries' economic growth, leading to a financial crisis. The low financial returns, high volatility in financial instruments, and stock market performance decrease caused many unprecedented challenges that has led the world economies into a global depression. The importance of supply chain in business operations and economic process remains viable in supporting financial trading. Organizational performance can be improved through buyer engagement in sustainable production to achieve social and environmental sustainability (Awan et al. 2020a, b). During the COVID-19 pandemic, the supply chain process was largely disrupted; the restrictions on free mobility, cessation of travel and transportation infrastructure, and financial trading led to unfortunate outcomes. Increased testing capacity and functional labs are essential to restore financial activities on a global scale. In some countries, testing capacity is limited due to untrained staff, shortage of healthcare professionals, inadequate healthcare infrastructure, and healthcare resources. The need for national and international collaboration, knowledge spillovers, and technology transfer to ensure healthcare resource supply to control the current pandemic is imperative. Hence, the need for full policy documents and implementation to manage the fall in the demand–supply gap. The developed country may play its active role in supporting the underdeveloped country's financial market through debt service suspension that can bring harmony to the developing countries during unprecedented times. These policy strategies will support the resumption of financial activities on a global scale.

The study has the following limitations that open the door for future studies to work on the stated theme to fill it with relevant factors. Firstly, the study used a single factor for assessing financial development, while it may add two to three more economic modeling factors for getting insights into financial sector reaction to the COVID-19 pandemic. Secondly, a list of a few other exogenous factors can be added, i.e., population density, per capita income, and real exchange rate, that create new empirical insights of the statistical inferences. Finally, the quantile regression technique can use for empirical testing that may show variations in the parameter estimates at different quantiles distribution. Hence, the greater need to study the risk of the COVID-19 pandemic on the global supply chain process can be projected in future studies.

## Data Availability

The data is freely available on World Development Indicator, published by World Bank on given URL ID: https://datacatalog.worldbank.org/dataset/world-development-indicators and Worldometer database, i.e., https://www.worldometers.info/coronavirus/
